# Ossicular Bone Damage and Hearing Loss in Rheumatoid Arthritis: A Correlated Functional and High Resolution Morphometric Study in Collagen-Induced Arthritic Mice

**DOI:** 10.1371/journal.pone.0164078

**Published:** 2016-09-30

**Authors:** Rensa Chen, Martin Schwander, Mary F. Barbe, Marion M. Chan

**Affiliations:** 1 Department of Microbiology and Immunology, Lewis Katz School of Medicine-Temple University, Philadelphia, PA, 19140, United States of America; 2 Department of Cell Biology and Neuroscience, Rutgers University, Piscataway, NJ, 08854, United States of America; 3 Department of Anatomy, Lewis Katz School of Medicine-Temple University, Philadelphia, PA, 19140, United States of America; University of South Florida, UNITED STATES

## Abstract

Globally, a body of comparative case-control studies suggests that rheumatoid arthritis (RA) patients are more prone to developing hearing loss (HL). However, experimental evidence that supports this hypothesis is still lacking because the human auditory organ is not readily accessible. The aim of this study was to determine the association between bone damage to the ossicles of the middle ear and HL, using a widely accepted murine model of collagen-induced arthritis (RA mice). Diarthrodial joints in the middle ear were examined with microcomputer tomography (microCT), and hearing function was assessed by auditory brainstem response (ABR). RA mice exhibited significantly decreased hearing sensitivity compared to age-matched controls. Additionally, a significant narrowing of the incudostapedial joint space and an increase in the porosity of the stapes were observed. The absolute latencies of all ABR waves were prolonged, but mean interpeak latencies were not statistically different. The observed bone defects in the middle ear that were accompanied by changes in ABR responses were consistent with conductive HL. This combination suggests that conductive impairment is at least part of the etiology of RA-induced HL in a murine model. Whether the inner ear sustains bone erosion or other pathology, and whether the cochlear nerve sustains pathology await subsequent studies. Considering the fact that certain anti-inflammatories are ototoxic in high doses, monitoring RA patients’ auditory function is advisable as part of the effort to ensure their well-being.

## Introduction

Rheumatoid arthritis is a systemic disease characterized by generalized inflammation and destruction of cartilage and bone. A primary symptomatic hallmark of RA is erosion of the arthrodial joints [1]. In humans, correlative studies reported that hearing loss occurs more frequently in RA patients than in normal individuals. These comparative case-control studies were conducted globally in the U.S., U.K., Spain, Italy, Japan, India, etc., and they covered over 900 RA subjects [2–14]. Children with juvenile chronic arthritis are also more susceptible to HL [15–18]. Contrarily, there are studies that dispute such a correlation [9, 19–22].

The air-filled middle ear contains the ossicles, which are three tiny bones (malleus, incus and stapes) linked by the incudomalleolar and incudostapedial diarthrodial joints [6, 23]. They serve to transmit sound-induced mechanical vibrations from the eardrum to the oval window of the fluid-filled cochlea [6]. Their bone tissue is susceptible to inflammatory injury [24], and damage to the ossicular chain is expected to impair sound conduction [25]. For example, chronic otitis and cholesteatoma can cause the destruction of incus and stapes, as well as conductive HL [26–28].

Here we tested the hypothesis that conductive HL is a common comorbidity in autoimmune arthritis using a well-established mouse model of collagen-induced arthritis. By micro-computer tomography imaging, we examined the morphology of the middle ear, and found a narrowing of the incudostapedial joint space and bone damage in RA mice. We also show that middle-ear sound transfer function is decreased, with auditory brain stem responses displaying altered absolute latencies for all waves. Altogether, the functional and structural findings are consistent with the presence of conductive HL.

## Methods

### Collagen-induced arthritis model

Arthritis was induced in 5 week old male DBA/1 mice (Jackson Laboratories, Bar Harbor, Maine) with 100 μg of chicken collagen II emulsified in complete Freund’s adjuvant and injected intradermally into the tail. The reaction was boosted with another injection of 50 μg of the collagen in incomplete Freund’s adjuvant at day 21 [29–32]. When the animals developed swelling in their paws, they were given extra thick bedding and easy reach to food and water. Hearing function was evaluated by recording the ABR before disease onset (pre-symptoms) on day 21–22 before booster, and when swelling had onset for at least 10 days, at day 48–50 [29]. After functional studies, the subjects were sacrificed by CO_2_ inhalation, compressed gas (100% CO_2_) and decapitation, temporal bones were harvested for morphometry by microCT. The protocol was approved by the Institutional Animal Care and Use Committees (IACUC) of Lewis Katz School of Medicine-Temple University and of Rutgers University before beginning of the study.

### ABR analysis

Hearing function in mice was evaluated by measuring the tone pip-evoked ABR with a RZ6 Multi-I/O processor (Tucker-Davis Technologies, TDT; Alachua, Florida) [33]. The acoustic stimuli were calibrated by coupling a 1/2″ ACO calibration microphone to the speaker tube via a 2 cc calibration syringe. The animals were examined for evidence of middle ear infection, effusion, and/or debris in the external auditory canals prior to recording. ABR recordings were conducted under sedation with 100 mg/kg ketamine hydrochloride and 2.4 mg/kg xylazine hydrochloride in a 37°C soundproof chamber. The active electrode was placed at the vertex, the reference electrode at the pinna and the ground electrode near the tail. A MF1 multi-field magnetic speaker (TDT) was coupled to the external ear canal. Mice were presented with tone pips at 4, 8, 12, 16 and 24 kilohertz (kHz) (12.1 tones per second, each 0.1 ms in duration), starting at 90 dB sound pressure level (SPL) and decreasing in 10 dB steps. The number of acquisition trials was set at 512 averages with 256 rarefaction and 256 condensation stimuli. BioSigRZ software was used to evaluate ABR waveforms and determine auditory thresholds (the lowest sound intensity which evokes a response), peak amplitudes and latencies. The results from ABR recordings of the treatment and control groups were averaged and compared statistically. This procedure has been documented in our publications [34]. Mice that displayed no ABR pattern at 90 dB (ABR threshold >90 dB) were considered deaf and those with increased thresholds (>20 dB) hearing impaired.

### MicroCT

High resolution morphometry of the bone structure of the ossicles was performed using a Skyscan-1170 microCT system (Bruker, Kontich, Belgium). Temporal bones were fixed in 4% buffered paraformaldehyde for 48 hours and then thoroughly washed and store in water until use. They were wrapped surrounded with water in Parafilm and placed in the Skyscan system. Samples were scanned 360 degrees around the vertical axis in rotation steps of 0.3 degrees. After an initial flat field correction, the following settings were used: 8.76 μm for camera pixel size, 300 nm for x-ray source spot size, 59 V for voltage, 167 μA for current, a 0.5 mm aluminum filter, a frame averaging of 3, and an image pixel size of 3 μm (which was also the voxel resolution). The duration of each scan was about 2 hours per specimen. Three dimensional images were reconstructed using image slices (tif files) in a cone-beam volumetric reconstruction software (Skyscan NRecon version 1.6.5) with the following settings: a ring artifact correction of 10, a beam hardening correction of 30%, and no smoothing. Post alignment correction was used as needed. The Skyscan CTAn program was used for 2D and 3D quantitative analysis of the incudostapedial joint and the stapes, to quantify the number [Po.N(cl)] and percent [Po(cl)] of closed pores per bone volume of interest. The percent of closed pores is defined as the percent of the total of solid plus closed pore volume within the volume of interest (i.e, the material porosity). The Skyscan CTVol, a surface-rendering program, was used to view and manipulate 3D models created in CTAn [35, 36]. For analyzing tissue mineral density, Houndsfield units of a set of calcium hydroxyapatite phantoms sized for mice bones, in water, were calculated and then used to compute the volumetric tissue mineral density of the segmented ossicles (which were filled with water) from the microCT slice images.

### Statistical analysis

Data distribution was tested for normality, variance and transformed if necessary, and then tested by parametric one way ANOVA to compare the ABR and microCT parameters in the arthritic and age-matched control mice. A *p* value of 0.05 was considered as significant. Sample sizes with statistical power to detect the effect with 95% confidence were used.

## Results

### Hearing in normal DBA/1 mice

**Fig 1:**
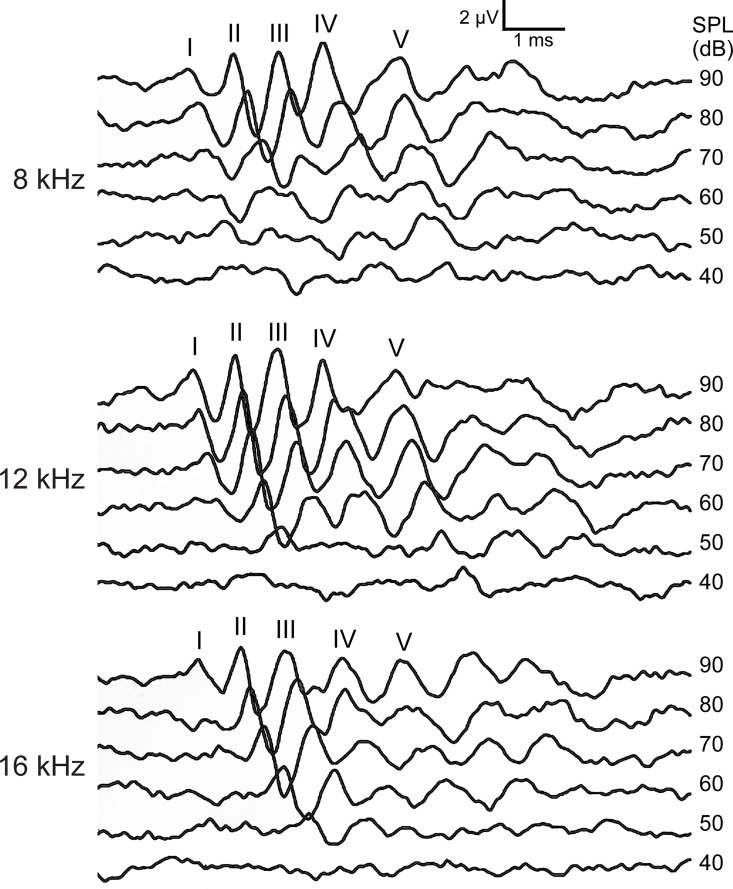
Analysis of auditory function in normal DBA/1 mice. Shown are the representative tone pip-evoked ABR waveforms from normal DBA/1 male mice at different frequencies (8–16 kHz). ABR waves (I-V) are indicated.

The study was initiated by determining the baseline hearing threshold levels of the normal DBA/1 mice. The murine collagen-induced arthritis model requires the use of the DBA/1 mouse strain, males in particular, to provide the genetics for the development of arthritis. While DBA/2 mice have been documented to develop age-related HL [37], hearing function of adult DBA/1 mice is normal. DBA/1 mice showed characteristic ABR waveforms with five waves (I-V) and sufficient hearing sensitivity within the age range used in the experiments (Fig 1) [34]. At 14 weeks of age, mice responded normally to tone bursts of 8, 12 and 16 kHz, albeit with higher ABR thresholds than some other strains, which is in agreement with the findings from previous studies by the Jackson Laboratory and Takeda *et al*. [38].

### Hearing thresholds in mice with induced arthritis

Immunized mice were considered positive for arthritis (RA mice) when one of their footpads had swollen by 15% or more [29]. All of the collagen-injected mice in this study showed footpad swellings great than 15% but their hind legs do not swell symmetrically to the same extent. The asymmetric nature is well recognized in the model. The cellular and bony damage in knees and metatarsophalangeal joints of the RA mice has been documented by histological and radiographic imaging in our previous publications [29, 30].

**Fig 2:**
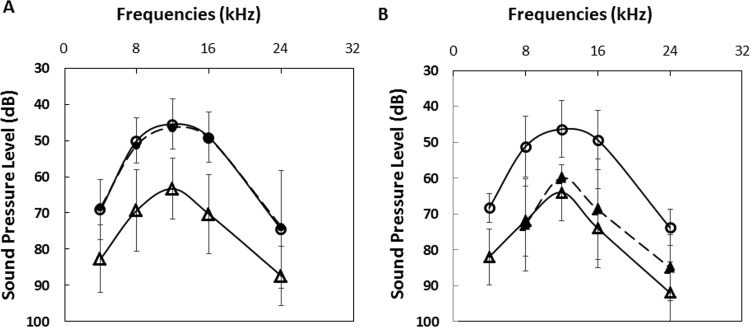
ABR threshold in RA mice. Panel [A] compares the ABR threshold in RA mice when arthritis was asymptomatic (dashed line with filled circles, n = 16, 32 ears) and after footpads had become swollen (solid line with open triangles, n = 13, 3 died, 26 ears). Panel [B] compares the hearing thresholds in mice of different RA severity. The dashed line with filled triangles shows the SPL of the group with footpad swelled by <50% (n = 9, 18 ears). The solid line-open triangles show the group that had footpads swollen by >50% (n = 4, 8 ears). In both panels, the solid lines with open circles indicate the threshold of the normal controls (n = 4, 8 ears). The plots and statistics included both ears.

**Fig 3:**
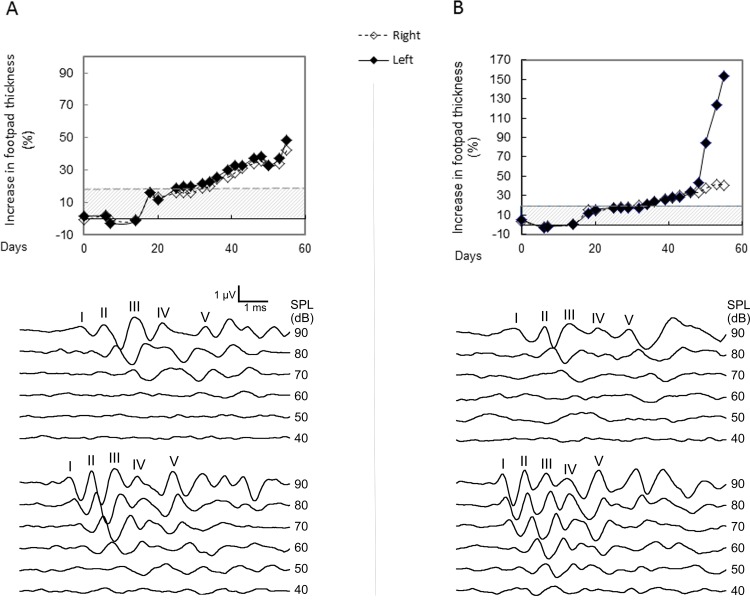
Comparison of joint swelling and HL severity in individual mice. The footpad swelling and pure tone-evoked ABRs elicited by pure tone bursts at 16 kHz in two representative RA mice. [A] is a RA mouse with footpads moderately swollen by less than 50%. [B] is one with a footpad severely swollen by 150%. The grey bar indicates the normal thickness of footpads of control mice which average is .076±0.004 mm. Arthritis is considered negative when footpad thickness had not increased by >15% and hearing is considered intact when auditory threshold is maintain within 20 dB of the baseline measurement. Both mice showed unilateral HL with sensitivity thresholds of about 70 dB. The ABR waveforms obtained from their unaffected ears (ABR threshold of about 50 dB) are shown in the respective lowest panels.

ABR analysis detected a decrease of hearing sensitivity in ears of the RA mice. Hearing thresholds were compared among the RA mice (1) before symptom development on day 21–22 after the first immunization, in 32 ears (2) on day 48–50, 10 days after footpads showed swelling subsequent to the booster, in 26 ears, and (3) in normal ears age-matched to when arthritis developed (Fig 2A). The hearing threshold of the controls (8 ears) at 8, 12, and 16 kHz were 50±6.3, 45 ±6.9, and 48±7.0 dB, respectively. Before symptom development, hearing thresholds of the immunized mice were similar to the controls. After the footpad became swollen, hearing thresholds increased by ≥ 20 dB across the frequencies in 46% of the 26 ears tested. Ears with sensitivity loss are then grouped according to whether they are from RA mice with footpads swollen by less than and or more than 50% (Fig 2B), that is swollen by 27%-43% (n = 9 mice) or have at least one footpad that was swollen by 73%-150% (n = 4 mice) at the height of inflammation, respectively. Despite the difference in RA manifestation, they did not differ in their degrees of HL. The sound pressure level (dB) thresholds of the two groups are similar at each frequency. Fig 3 shows the footpad measurements and ABR waveforms at 16 kHz of two representative mice.

Analysis of individual mice revealed that hearing impairment manifested in all RA mice, but some mice (71%) were affect unilaterally only in one not bilaterally in both ears. Asymmetry in the footpad swelling is also a phenomenon that has been observed in the animal model and human RA patients [9, 10, 18]. The HL, furthermore, did not parallel the severity or correspond laterally to arthritis in the limbs. There is currently no verified explanation available as to why hearing loss is asymmetrical or why unilateral manifestation is more prevalent. The findings that the ears and legs are not affected equally or in pairs, nonetheless, reflect that each joint is affected independently in this systemic disease. It should also be noted that low level inflammation and hearing loss, below the detection threshold, might be present in the ear that is classified as unaffected.

### 3-D micro-computer tomography of the ossicles

**Fig 4:**
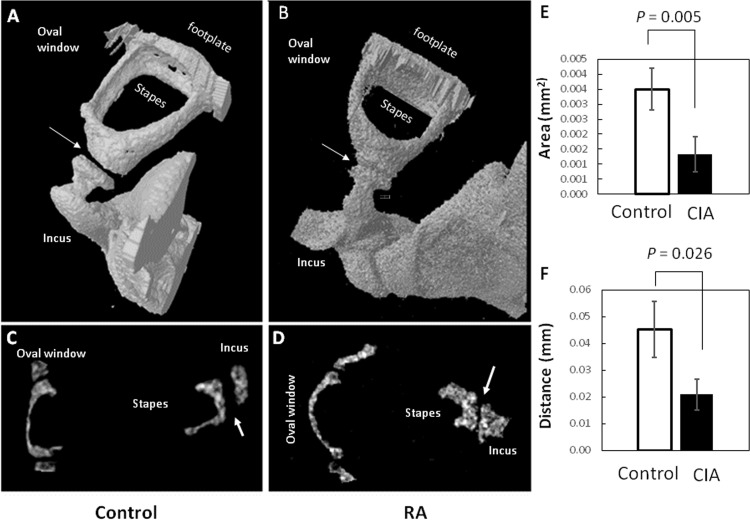
Joint space narrowing. 3-dimensional, [A] & [C], and 2-dimensional, [B] & [D], reconstruction micro-CT images of typical incudostapedial joints (arrows) from control (n = 3 ears) and RA mice (n = 3 ears). The image pixel size was 3 microns and rendering was done with the lower grey threshold set at 110 and the upper grey threshold at 225. To the right are numerical comparisons of the total area [E] and linear distance [F] of the space between the incus and stapes of the two groups. These spaces were measured using the Skyscan CTAn program, by marking three positions: the center and the two edges of the sections with the widest gaps.

**Fig 5:**
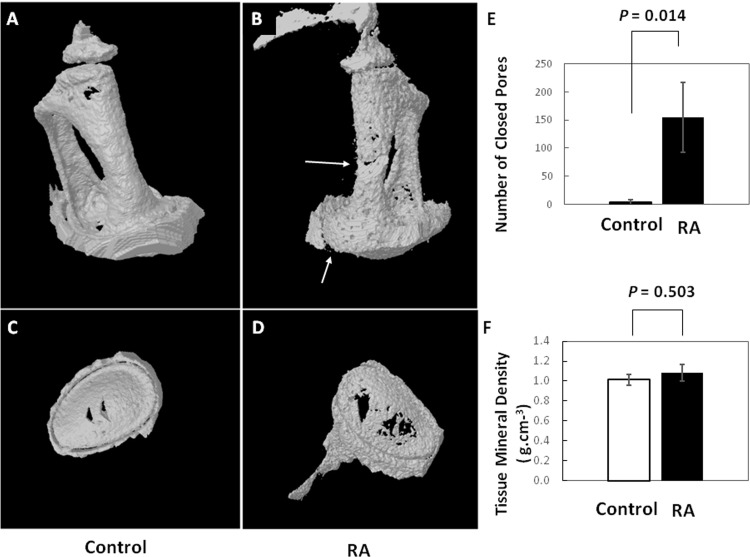
Altered microarchitecture of anterior crus and footplates of RA mice. Shown are 3-dimensional reconstruction micro-CT images of the stapes [A] and footplates [C] in typical control (n = 3 ears) and stapes [B] and footplates [D] (n = 3 ears) of RA mice. Note the erosion/roughness of the surfaces, enlarged holes in the footplate, and closed pores internal in cutout regions of the crus and footplates (arrows) in the RA mice. MicroCT parameters have been described above in Fig 4. The graphs compare the number of closed pores [E] and tissue mineral density [F] in the stapes, respectively (n = 3 ears). Tissue mineral density is defined as the volumetric density of known calcium hydroxyapatite phantoms.

*Ex vivo* bone morphometric analysis was performed on ears of mice with hearing threshold of higher than 70 dB at 16 KHz, 20 dB or more above the normal threshold. Using high-resolution microCT, the ossicles were examined within the temporal bones (Fig 4). Fig 4A & 4B show the 3D structure of the incudostapedial joint from representative control and RA mice. As measured using the 2D cross-sectional image (Fig 4C & 4D), the distance and area of space between the incus and stapes was decreased by more than half in the RA group (Fig 4F). To compare the microarchitecture of the bones, we chose the stirrup-shaped stapes, which allows the region of interest (ROI) to be most precisely defined (Fig 5A–5D). The stapes from RA mice had hundreds of closed pores, averaging 155, which occupied 12% of the volume of these cortical bones. By comparison, the controls had less than 10 closed pores comprising only 0.4% of their volume (Fig 5E). The tissue mineral density in the two groups, nonetheless, was similar: 1.086±0.08358 g.cm^3^ and 1.014±0.0529 g.cm^3^. Thus, calcium hydroxyapatite was maintained in the mineralized bone of the stapes (Fig 5F). This combination of bone morphometric characteristics is typically indicative of osteoclastic resorptive spaces and vascular profiles, as previously described by Massicotte *et al*, 2015 [39].

### Nature of HL

**Fig 6:**
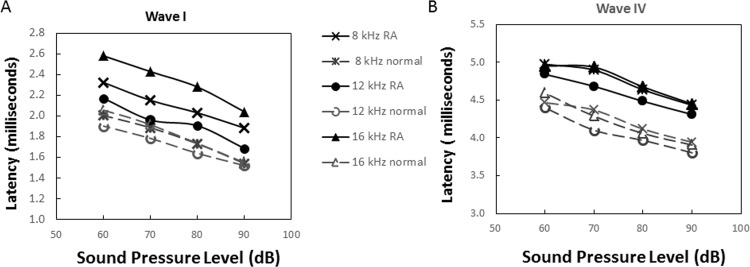
Tone pip-generated wave I and IV latency-intensity functions. The time between the onset of the tone pip and the peak of each ABR wave was determined. Presented are the latency-intensity function of RA mice (solid lines with filled markers) and normal controls (dashed lines with open markers) for waves I [A] and IV [B]. The average latency of RA (n = 18 ears) and normal mice (n = 7 ears) for tone pips at 8 kHz (crosses, X), 12 kHz (circles, O) and 16 kHz (triangles, Δ) were plotted against the stimulus intensity (dB).

**Fig 7:**
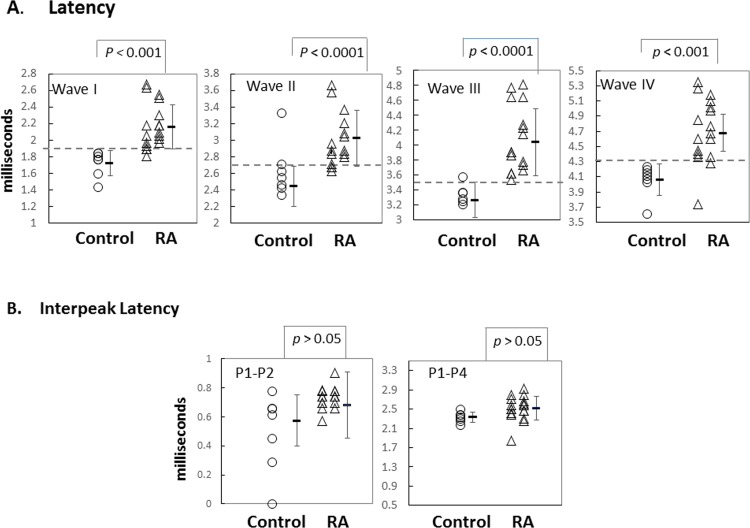
Ear by ear comparison of absolute and interwave latencies at 16 kHz and 80 dB. Panel [A] shows the absolute latencies of waves I-IV for tone pips at 16kHz. Each dot represents one ear. The averaged latencies and standard deviations for each group are indicated by black lines and error bars, respectively. Panel [B] shows the interpeak latencies of waves I-II and I-IV for the two groups of mice in response to 16 kHz tone pip stimuli. The number of ears analyzed in the groups is described in the text.

The nature of the HL was investigated by analyzing the ABR waveforms. Comparisons were made between the absolute latencies of RA versus control mice across 8-12-16 kHz, from 60–90 dB, and for ABR waves I and IV. The absolute latencies for both waves were increased in the RA group compared to the control group but latency shifts were similar at all sound intensities analyzed (Fig 6). Then, a comparison was performed on the RA and control groups for each of the four waves but focusing on 16 kHz at 80 dB (Fig 7). The RA group’s average latency was 2.29±0.26 milliseconds (ms) for wave I (n = 18 ears), 3.02±0.33 ms for wave II (n = 18), 4.04±0.45 ms for wave III (n = 18), and 4.76±0.42 ms for wave IV (n = 19). The control group’s average latency was 1.98±0.21 ms for wave I (n = 8 ears), 2.44±0.24 ms for wave II (n = 8), 3.27±0.33 ms for wave III (n = 7) and 4.06±0.24 ms for wave IV (n = 7). These differences in absolute latencies between the two groups were highly significant, at 99% or 99.9% confidence, for the all four waves. However, the interpeak latencies (waves I–II and waves I-IV) were similar in the two groups (*p*>0.05). Additionally, the ABR waveforms show that the delay in latency were similar when the stimulus level was altered (Table 1). In combination, the threshold increase and absolute latency pattern change is consistent with an impediment in the conduction of sound through the middle ear, even though we cannot exclude the presence of a sensorineural pathology.

**Table 1:** Pattern shift in ABR waves.

		Waves:	I		II		III		IV	
			ms	*p* value	ms	*p* value	ms	*p* value	ms	*p* value
**RA**	90–70 dB	Ave	**0.46**		**0.50**		**0.72**		**0.71**	
n = 28		SD	0.24		0.22		0.39		0.14	
				0.45		0.72		0.38		0.63
	80–60 dB	Ave	**0.30**		**0.55**		**0.71**		**0.74**	
		SD	0.65		0.24		0.27		0.34	
**Control**	90–70 dB	Ave	**0.39**		**0.47**		**0.62**		**0.63**	
n = 7		SD	0.18		0.15		0.29		0.41	
				0.85		0.30		0.80		0.70
	80–60 dB	Ave	**0.35**		**0.55**		**0.74**		**0.81**	
		SD	0.28		0.15		0.24		0.56	

Table 1 displays the difference in latency of the ABR waveform peaks elicited by 16 kHz tone pip-stimuli at two different sound levels. Average absolute latencies for wave I, wave II, wave III, or wave IV at different stimulus intensities (dB) were determined for the RA and the control group. The differences in latency (lapse) between 90 and 70 dB and between 80 and 60 dB were derived for each wave and each ear and then averaged (bold numbers). The number of ears analyzed in each group (n) and standard deviations (SD) are indicated. The *p* values compare the lapse from 90 to 70 dB versus the lapse from 80 to 60 dB within the RA and, similarly, within the control groups. *P* > 0.05 indicates that the intervals are not significantly different.

## Discussion

While a series of human studies support the hypothesis that patients with RA are more prone to HL, the pathogenesis of HL in RA is still controversial. HL in these cases has been attributed to lesions in the middle ear [6, 12, 15, 16] and inner ear [35, 40, 41]. The suspected etiologies include ossicular stiffness and discontinuity [6, 11], immune complexes-mediated vasculitis [8, 10], and destruction by antibody targeting the inner ear [14]. HL in RA patients has been described as conductive [10, 21, 42], sensorineural [7, 8, 13, 14, 19], or mixed [2, 9, 11, 18, 43]. These premises, nevertheless, were inferred from impaired hearing functions detected with different audiometric tests, and the studies were disparate in gender, as well as age. In addition, the potential contribution of sensorineural HL-inducing immunomodulatory drugs, salicylates and non-steroidal anti-inflammatory drugs (NSAIDs), used in the treatment of RA patients precludes a straightforward interpretation of the data from the literature.

The cardinal pathology of RA is the destruction of articular joints. However, whether the diathroses joints of the middle ear are susceptible to the disease process remains controversial. In humans, the auditory organ is not easily accessible for structural analysis. Using a well-established murine model for RA, we have examined the temporal bone for structural damage in the ossicles and integrated these findings with ABR data to investigate whether there is a correlation between structural changes and hearing impairment.

Osteoclast-mediated resorption (formation of Howships lacunae) is one of the characteristics of inflammation of the compact bone (Haversian systems) in the middle ear of rodents [24]. Our study shows RA leads to erosive degradation of bone microarchitecture in the middle ear and in parallel hearing sensitivity in these mice was decreased by 20 dB or more. The efficiency for sound transmission velocity was affected across all intensities in a manner that is consistent with conductive HL. In parallel, microCT radiography analysis revealed that the incudostapedial joint space have narrowing in the RA mice. Furthermore, hundreds of pores and pits were formed at their stapes. Erosive degradation of bone microarchitecture in the middle ear might be the cause of the auditory phenotype in RA mice. We did not detect changes in mineral density of the remaining bone matrix. Possibly, the cortical bones of the ossicles, unlike the phalanges which have trabecular bones, are less susceptible to mineral loss [44].

Salomonsen *et al*. (2010) observed osteonogenesis and new bone deposition, which may lead to fixation, simultaneous with resorption in inflammation induced by bacteria-mediated acute otitis media [24]. Discontinuity of the ossicular chain leads to loss of mechanical amplification. In mice, interrupting the incudo-stapedial joint or fixing the ossicular chain cause up to 25 dB increase in hearing threshold [45]. Our study cannot conclude whether there was immobilization of the ossicles by bony fixation. However, visual examination of the 3-D micro-CT model revealed contact between footplates to oval windows at part of the circumference in samples from RA mice. Histological analyses will likely show the cellular mechanism of ossicular damage accounting for the formation of pores and reveal the presence of bone sclerosis, if any. The likelihood that compromised mobility might be present and that prolongation of damage and reparation might lead to otosclerosis in our RA mice exists.

Further study with archival temporal bones from RA donors with a family history of hearing loss will show whether similar damage occur in humans. In humans, stapes fixations can produce 40 dB conductive hearing loss [46] and bilateral conductive HL have been demonstrated in RA patients by tympanometric analysis [12]. Unfortunately, tympanometry for mice is only available to a few investigators and our subjects could not be further examined by this test for middle ear function in humans.

Comparing the effects of RA-induced bone damage in mice and humans, our study reveals that the two are similar in that HL is detected after symptom onset but the degree of HL does not parallel the activity or duration of manifestation in the skeletal joints [11]. We proposed that the organs are affected independently in this systemic disease, but it is also possible that each subject’s disposition to conductive HL is predetermined. Once the amplification function of the ossicule chain has been maximally compromised, there is no further increase in ABR thresholds with higher degree of inflammation. Conversely, our findings differ from several human studies in that every mouse with collagen-induced arthritis had HL, whereas several clinical studies reported HL in 30–40% of RA sufferers [5, 8, 9, 11, 12, 13]. This may be due to the heterogeneity of the human population. Nevertheless, neither the mice nor humans became profoundly deaf; thus, explaining why the RA-associated HL has been reported as subclinical and is clinically underappreciated.

The structural changes, increase in hearing threshold and delayed latencies in ABR are evidence for conductive impairment consistent with conductive HL. However, loss of sound amplification in the middle ear may not be the only cause of the HL. Sensorineural HL, originating in the inner ear or auditory nerve, has been reported in type II collagen-induced autoimmune ear diseases of rats, guinea pigs, and mice [38, 47, 48]. Our study did not include an examination of the inner ear. Thus, whether the cochlear bone (bony labyrinth) and the hair cells are also damaged await the histological analysis of these structures in human temporal bones and sensorineural transmission analysis by distortion products otoacoustic emission (DPOAE) testing.

Currently, RA patients are treated without otological monitoring and care. Our study of the murine collagen-induced arthritis, a widely recognized model for human RA, corroborates the clinical observations that HL is a co-morbidity of RA. Considering the low reparative capacity of the auditory bony structure and, the fact that many RA patients regularly take ototoxic anti-inflammatories, such as salicylates [49, 50], early management of middle ear inflammation might be advantageous to prevent HL. This study supports the incorporation of audiometric evaluation in rheumatoid arthritis patients. It should be noted that conductive HL has also been associated with other autoimmune and inflammatory joint diseases, such as ankylosing spondylitis and Lyme disease [51, 52].

## Abbreviations

ABR: auditory brain stem response
CHL: conductive hearing loss
CIA: collagen-induced arthritis
dB: decibel
HL: hearing loss
kHz: kilohertz
microCT: micro-computer tomography
ms: milliseconds
RA: rheumatoid arthritis
SD: standard deviation
SPL: sound pressure level
